# Astrocyte elevated gene-1 regulates osteosarcoma cell invasion and chemoresistance via endothelin-1/endothelin A receptor signaling

**DOI:** 10.3892/ol.2012.1056

**Published:** 2012-12-03

**Authors:** BO LIU, YI WU, DAN PENG

**Affiliations:** Department of Orthopaedics, Second Xiangya Hospital, Central South University, Changsha, Hunan 410011, P.R. China

**Keywords:** astrocyte elevated gene-1, endothelin-1, endothelin A cell invasion, chemoresistance, osteosarcoma, phosphatidylinositol 3-kinase, receptor

## Abstract

Astrocyte elevated gene-1 (AEG-1) and endothelin-1 (ET-1)/endothelin A receptor (ETAR) signaling have been demonstrated to be important in osteosarcoma (OS) progression. In the present study, we explored the interaction between AEG-1 and ET-1/ETAR signaling in OS cells, and investigated the mechanism(s) through which the functional interaction may impact OS cell invasion and chemoresistance. Overexpression and knockdown of AEG-1 were performed in Saos-2 and MG-63 OS cells, respectively. Overexpression of AEG-1 in Saos-2 cells significantly increased ET-1 expression (at both the mRNA and protein levels), cell invasion, MMP-2 expression and cell survival against cisplatin. These effects were eradicated using a selective phosphatidylinositol 3-kinase (PI3K) inhibitor, LY294002, or a selective ETAR inhibitor, BQ123. Knockdown of AEG-1 in MG-63 cells significantly decreased ET-1 expression (at both the mRNA and protein levels), cell invasion, MMP-2 expression and cell survival against cisplatin. Exogenous ET-1 restored cell invasion and MMP-2 expression levels in MG-63 cells, in which AEG-1 had been knocked down, in the presence of LY294002, but not in the presence of BQ123. However, exogenous ET-1 only partially rescued cell survival against cisplatin-induced apoptosis in the presence of LY294002, in cells in which AEG-1 had been knocked down. In conclusion, we have demonstrated that AEG-1 regulates ET-1 expression at the transcriptional level in a PI3K-dependent manner in OS cells. Downstream of PI3K, ET-1/ETAR signaling primarily mediates the promoting effect of AEG-1 on OS cell invasion, likely through the upregulation of MMP-2 expression, thus, ET-1/ETAR signaling partially, but significantly, mediates the AEG-1-induced chemoresistance in OS cells. To the best of our knowledge, this study has provided the first evidence of a functional association between AEG-1 and ET-1/ETAR signaling in OS cells, which adds novel insights into the molecular mechanism of OS metastasis and chemoresistance.

## Introduction

Osteosarcoma (OS) is the most frequent primary bone malignancy and the eighth most common type of cancer among children, comprising 2.4% of all malignancies in pediatric patients and ∼35% of all types of bone cancer (1). OS is characterized by high local aggression and a tendency to metastasize to the lungs and distant bones. For patients with localized forms of OS, the recovery rate is ∼65%. For those who present with metastases at the time of diagnosis, the survival rate is 25% (2,3). Thus, it is important to identify and confirm potential therapeutic targets involved in OS progression.

Astrocyte elevated gene-1 (AEG-1), also known as metadherin (MTDH), is a multifunctional oncogene. This gene is overexpressed in a variety of types of human cancer, although it was originally isolated as a novel HIV-1- and TNFα-induced transcript from primary human fetal astrocytes (4,5). As a downstream target of Ha-Ras, AEG-1 is important in regulating tumorigenesis, invasion, metastasis and angiogenesis (6). In a study by Wang *et al,* AEG-1 was found to be overexpressed in OS tissues, and the overexpression of AEG-1 strongly correlates with OS metastasis and poor survival (7). The data suggest that AEG-1 is important in OS progression via matrix metalloproteinase 2 (MMP-2), and that AEG-1 may be a useful biomarker for the prediction of OS progression and prognosis (7).

Endothelin-1 (ET-1) is expressed in a variety of malignancies, and promotes tumor cell proliferation and survival through the ET A receptor (ETAR) (8). ET-1 and ETAR are expressed in OS cells and tissue (9,10). Felx *et al* revealed that ET-1 may promote OS cell invasion by inducing the synthesis of MMP-2 through ETAR, suggesting an important role of ET-1 in OS metastasis (9). *In vitro* studies have demonstrated that blocking ETAR leads to the inhibition of OS cell invasion, suggesting that ETAR is a potential therapeutic target for OS metastasis (9,10).

In the present study, we conducted the first investigation into the interaction between AEG-1 and ET-1/ETAR signaling in OS cells, and assessed how the functional interaction may impact OS cell invasion and survival against chemotherapy agents.

## Materials and methods

### Cells lines, plasmids and reagents

Saos-2 and MG-63 human OS cell lines were purchased from the American Type Culture Collection (Rockville, MD, USA). Human AEG-1 cDNA was subcloned into a pcDNA 3.1 expression vector. AEG-1/MTDH (sc-77797-V) short hairpin RNA (shRNA) lentiviral particles, control shRNA lentiviral particles-A (sc-108080), and anti-ET-1 (sc-21625) and anti-MMP-2 (sc-10736) antibodies were purchased from Santa Cruz Biotechnology, Inc. (Santa Cruz, CA, USA). Anti-AEG-1 antibody (HPA010932) was purchased from Sigma (St. Louis, MO, USA). All secondary antibodies were purchased from Jackson ImmunoResearch Laboratories, Inc. (West Grove, PA, USA). The ET-1 enzyme-linked immunosorbent assay (ELISA) kit was purchased from R&D Systems (Minneapolis, MN, USA). The DeadEnd™ Fluorometric terminal deoxynucleotidyl transferase mediated nick-end labeling (TUNEL) system was purchased from Promega (Madison, WI, USA). Superfect™ transfection reagent was purchased from Qiagen (Valencia, CA, USA). Puromycin, cisplatin, synthetic ET-1, LY294002, BQ123 and reagent grade chemicals were purchased from Sigma.

### Real-time quantitative reverse transcription (RT)-PCR

RNA was prepared from brain tissue samples using the TRIzol reagent followed by purification with the TURBO DNA-free system (Ambion; Austin, TX, USA). SuperScript II reverse transcriptase (Invitrogen; Carlsbad, CA, USA) was used to synthesize cDNA. Real-time quantitative PCR was performed in the LightCycler thermal cycler system (Roche Diagnostics; Indianapolis, IN, USA) using the SYBR-Green I kit (Roche Diagnostics) as per the manufacturer’s instructions. Results were normalized against those of the housekeeping gene glyceraldehyde-3-phosphate dehydrogenase (*GAPDH*) in the same sample. The primer sequences used were as follows: Forward: 5′-TCCTCTGCTGGTTCCTGACT-3′ and reverse: 5′-CAGAAACTCCACCCCTGTGT-3′ for human *ET-1*; forward: 5′-GACTCATGACCACAGTCCATGC-3′ and reverse: 5′-AGAGGCAGGGATGATGTTCTG-3′ for human *GADPH*. Each experiment was repeated twice and performed in triplicate.

### Transfection and lentiviral transduction

The AEG-1 expression construct was transfected into Saos-2 cells using the Superfect transfection reagent according to the manufacturer’s instructions. Pools of stable transductants were generated via selection with puromycin (5 *μ*g/ml) according to the manufacturer’s protocol. The AEG-1/MTDH shRNA lentiviral particles contained expression constructs encoding target-specific 19–25 nt, as well as hairpin, shRNA designed to specifically knockdown AEG-1 gene expression. The control shRNA lentiviral particles contained a scrambled shRNA sequence that is not capable of initiating the degradation any cellular mRNA, and were used as negative controls for AEG-1/MTDH shRNA lentiviral particles. Lentiviral transduction was performed in Saos-2 and MG-63 cells. Pools of stable transductants were generated via selection with puromycin (5 *μ*g/ml) according to the manufacturer’s protocol (Santa Cruz Biotechnology, Inc.).

### In vitro cell invasion assay

Transwell^®^ cell invasion assays (Corning Life Sciences; Lowell, MA, USA) were performed as previously described (23). Briefly, Transwell cell-culture chambers (pore size, 8 *μ*m; BD Biosciences; Bedford, MA, USA) for 24-well plates were coated with 50 *μ*l Matrigel (10 mg/ml; BD Biosciences) diluted 1:3 in Roswell Park Memorial Institute (RPMI)-1640 medium. Saos-2 and MG-63 cells were seeded in the upper chamber at 5×10^5^ cells/well in RPMI-1640 serum-free medium. Complete medium (600 ml) was added to the lower chamber. Cells were treated with ET-1 (10 or 100 pM) and/or LY294002 (50 *μ*M) or BQ123 (5 *μ*M) and allowed to migrate for 24 h followed by fixation and staining with crystal violet. Migrated cells were counted in 10 random fields per chamber under a microscope. Each experiment was repeated three times and conducted in triplicate.

### Immunoassays

Secreted ET-1 levels in cell culture supernatants were determined using an ET-1 ELISA kit. In brief, cells were grown to confluence in 10-cm dishes in RPMI-1640 medium supplemented with 10% fetal bovine serum (FBS) in a humidified atmosphere of 95% air and 5% CO_2_ at 37°C.. The medium was then replaced with serum-free medium and cells were further incubated for 16 h. Cell culture supernatants were collected for ELISA according to the manufacturer’s instructions (R&D Systems). ELISA-detected ET-1 concentrations were normalized against the cell number (per 10^6^ cells) and are shown as the fold change relative to that of the normal control cells (designated as 1). Each ELISA experiment was repeated three times and performed in duplicate. For western blot analyses, protein was extracted by a lysis buffer containing 150 mM NaCl, 2% Triton X-100, 0.1% SDS, 50 mM Tris (pH 8.0) and 10% protease inhibitor cocktail (Sigma), and stored at −20°C. Equal amounts of protein (25 *μ*g) for each sample were loaded into pre-cast 7.5% Mini Protean TGX gels (BioRad; Hercules, CA, USA) and separated by electrophoresis for 50 min at 200 V. The separated proteins were transferred onto a polyvinylidene fluoride (PVDF) transfer membrane (Amersham Biosciences/GE Healthcare; Piscataway, NJ, USA) for 55 min at 100 V. Membranes were incubated for 1 h with a 1:500 dilution of anti-AEG-1, anti-MMP-2 or anti-ET-1 antibody, and then washed and revealed using secondary antibodies with horseradish peroxidase conjugate (1:5000; 1 h). Peroxidase activity was revealed using a GE Healthcare ECL kit. Proteins were quantified prior to being loaded onto the gel, and equal loading of protein was verified by Ponceau coloration.

### Measurement of apoptosis by TUNEL assay

The TUNEL assay was performed using the DeadEnd Fluorometric TUNEL system according the manufacturer’s instructions. Cells were treated with cisplatin (10 nM) in the presence or absence of ET-1 (10 or 100 pM) and/or LY294002 (50 *μ*M) or BQ123 (5 *μ*M) for ≤8 h. Apoptotic cells exhibited a green nuclear fluorescence that was detected using a standard fluorescein filter. Cells stained with 4′,6-diamidino-2-phenylindole (DAPI) exhibited a blue nuclear fluorescence. Slides were observed under fluorescence microscopy with the relative number of apoptotic cells determined by counting the number of TUNEL-positive cells in five random fields (magnification, ×100) for each sample.

### Statistical analysis

Statistical analyses were performed with the Statistical Package for the Social Sciences (SPSS) software for Windows, version 10.0. Data values were expressed as the mean ± SD. Comparisons of means among multiple groups were performed with one-way ANOVA followed by *post hoc* pairwise comparisons using the least significant difference method. The significance level of this study was set at a two- tailed α=0.05.

## Results

### Effect of overexpression and knockdown of AEG-1 on ET-1 expression in OS cells

**Figure 1.**
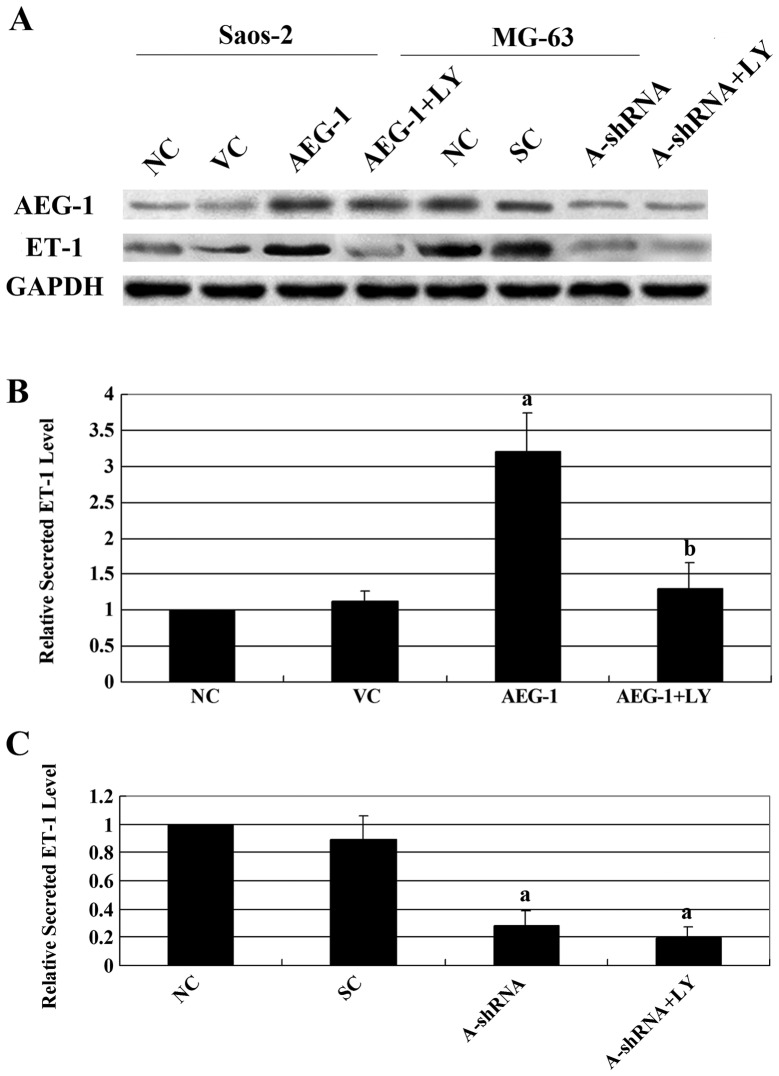
Western blot analysis of astrocyte elevated gene-1 (AEG-1) and endothelin-1 (ET-1) expression in Saos-2 and MG-63 osteosarcoma (OS) cells. (A) In Saos 2 cells, the expression of AEG-1 in normal control cells (NC), cells stably transfected with empty pcDNA3 vector (VC) and cells stably transfected with pcDNA3-AEG-1 expression vector (AEG-1) with or without LY294002 (LY; 50 *μ*M) treatment were analyzed with western blot analysis. In MG-63 cells, the expression of AEG-1 in NC, cells stably transduced with scrambled control shRNA (SC) and cells stably transduced with AEG-1-shRNA (A-shRNA) with or without LY (50 *μ*M) treatment were analyzed with western blot analysis. GAPDH blotting was used as a loading control. The secreted ET-1 levels in cell culture supernatants for the above experimental groups in (B) Saos-2 and (C) MG-63 cells were quantified using ELISA and normalized against the cell number (per 10^6^ cells). The secreted ET-1 level is shown as the fold change relative to that of NC (designated as 1). ^a^P<0.05 compared with (B) NC and VC or (C) SC; ^b^P<0.05 compared with AEG-1. ShRNA, short hairpin RNA.

Saos-2 cells were found to exhibit a relatively low constitutive AEG-1 expression compared with MG-63 cells (Fig. 1). Thus, to investigate the interaction between ET-1 and AEG-1 in OS cells, Saos-2 cells were stably transfected with an AEG-1 expression vector to induce AEG-1 overexpression, while MG-63 cells were stably transfected with AEG-1-shRNA to knock down AEG-1. Compared with the controls, AEG-1 was overexpressed by >3-fold in Saos-2 cells, and the endogenous AEG-1 level was knocked down by >70% in MG-63 cells. ET-1 was detected at a lower constitutive level in Saos-2 cells compared with that of MG-63 cells. In Saos-2 cells, overexpression of AEG-1 increased the ET-1 level by >2-fold compared with the controls. This effect was eradicated by the addition of the selective phosphatidylinositol 3-kinase (PI3K) inhibitor, LY294002. In MG-63 cells, knockdown of AEG-1 decreased the level of ET-1 by >2-fold compared with the controls, while treatment with LY294002 demonstrated no more significant effects. Similar results were observed at the secreted ET-1 level in the two cell lines, suggesting that AEG-1 signaling regulates ET-1 expression in a PI3K-dependent manner in OS cells.

**Figure 2.**
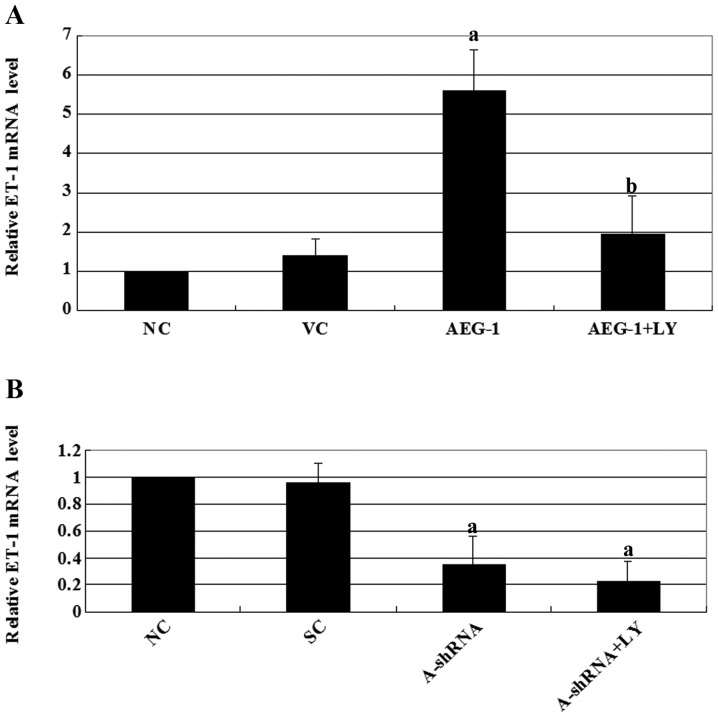
Endothelin-1 (ET-1) mRNA level in Saos-2 and MG-63 cells. (A) In Saos-2 cells, the ET-1 mRNA level in normal control cells (NC), cells stably transfected with empty pcDNA3 vector (VC) and cells stably transfected with pcDNA3-AEG-1 expression vector (AEG-1) with or without LY294002 (LY; 50 *μ*M) treatment were analyzed with real-time reverse transcription (RT)-PCR. (B) In MG-63 cells, the ET-1 mRNA level in NC, cells stably transduced with scrambled control shRNA (SC) and cells stably transduced with AEG-1-shRNA (A-shRNA) with or without LY (50 *μ*M) treatment were analyzed with real-time RT-PCR. The ET-1 mRNA level is shown as the fold change relative to that of NC (designated as 1). ^a^P<0.05 compared with (A) NC and VC or (B) SC; ^b^P<0.05 compared with AEG-1. ShRNA, short hairpin RNA.

Real-time RT-PCR revealed that overexpression of AEG-1 in Saos-2 cells increased the ET-1 mRNA level by >4-fold compared with the controls. This effect was eradicated by adding LY294002 (Fig. 2A). By contrast, knockdown of AEG-1 in MG-63 cells decreased the ET-1 mRNA level by ∼3-fold compared with the controls, while treatment with LY294002 demonstrated no significant further effects (Fig. 2B). The results indicate that AEG-1 signaling regulates ET-1 expression at the transcriptional level in a PI3K-dependent manner in OS cells.

### Effect of overexpression and knockdown of AEG-1 on OS cell invasion and MMP-2 expression

**Figure 3.**
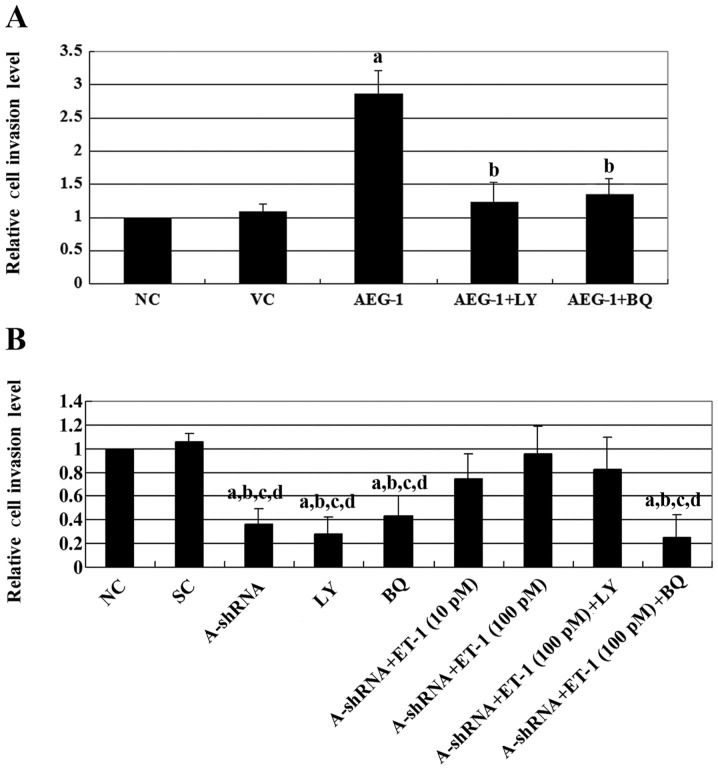
*In vitro* cell invasion in Saos-2 and MG-63 cells. (A) In Saos-2 cells, *in vitro* cell invasion assays were performed in normal control cells (NC), cells stably transfected with empty pcDNA3 vector (VC), and cells stably transfected with pcDNA3-AEG-1 expression vector (AEG-1) with or without LY294002 (LY; 50 *μ*M) or BQ123 (BQ; 5 *μ*M) treatment. (B) In MG-63 cells, *in vitro* cell invasion assays were performed in NC, cells stably transduced with scrambled control shRNA (SC), and cells stably transduced with AEG-1-shRNA (A-shRNA) with or without ET-1 (10 pM and 100 pM) treatment alone or ET-1 (100 pM) combined with LY (50 *μ*M) or BQ (5 *μ*M). Cells treated with LY (50 *μ*M) or BQ (5 *μ*M) alone were also analyzed. Invasion cell numbers were counted and the cell invasion level is shown as the fold change in invasion cell number relative to that of NC (designated as 1). ^a^P<0.05 compared with (A) NC and VC or (B) SC; ^b^P<0.05 compared with (A) AEG-1 or (B) A-shRNA and ET-1 (10 pM) ; ^c^P<0.05 compared with A-shRNA and ET-1 (100 pM); ^d^P<0.05 compared with A-shRNA, ET-1 (100 pM) and LY. ShRNA, short hairpin RNA.

**Figure 4.**
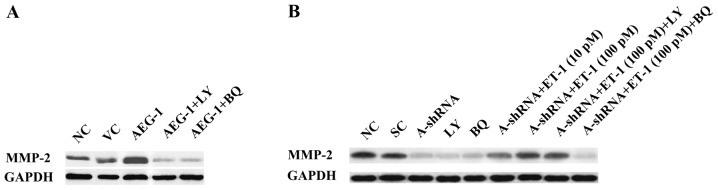
Western blot analysis of matrix metalloproteinase-2 (MMP-2) expression in Saos-2 and MG-63 cells. (A) In Saos-2 cells, the expression of MMP-2 in normal control cells (NC), cells stably transfected with empty pcDNA3 vector (VC), and cells stably transfected with pcDNA3-AEG-1 expression vector (AEG-1) with or without LY294002 (LY; 50 *μ*M) or BQ123 (BQ; 5 *μ*M) treatment were analyzed with western blot analysis. (B) In MG-63 cells, the expression of MMP-9 in NC, cells stably transduced with scrambled control shRNA (SC), and cells stably transduced with AEG1-shRNA (A-shRNA) with or without ET-1 (10 pM and 100 pM) treatment alone or ET-1 (100 pM) combined with LY (50 *μ*M) or BQ (5 *μ*M) were analyzed with western blot analysis. Cells treated with LY (50 *μ*M) or BQ (5 *μ*M) alone were also analyzed. GAPDH blotting was used as a loading control. ShRNA, short hairpin RNA.

Both AEG-1 and ET-1 have been demonstrated to promote OS cell invasion through MMP-2 (7,9). To investigate the effect of the interaction between AEG-1 and ET-1/ETAR signaling on OS invasion, we performed *in vitro* cell invasion assays and examined the MMP-2 expression level in the two cell lines. Overexpression of AEG-1 in Saos-2 cells increased cell invasion by ∼2.5-fold compared with the controls (Fig. 3). This effect was eradicated by the addition of either LY294002 or the ETAR inhibitor, BQ123. By contrast, knockdown of AEG-1 in MG-63 cells decreased cell invasion by >2-fold compared with the controls. Treatment with exogenous ET-1 increased cell invasion in a dose-dependent manner in cells in which AEG-1 had been knocked down. This effect was blocked by BQ123, but not by LY294002. Similar results were observed with MMP-2 expression (Fig. 4). The results suggest that AEG-1 promotes OS cell invasion primarily through ET-1/ETAR, which functions downstream of PI3K and regulates MMP-2 expression.

### Effect of overexpression and knockdown of AEG-1 on OS cell survival against cisplatin-induced apoptosis

**Figure 5.**
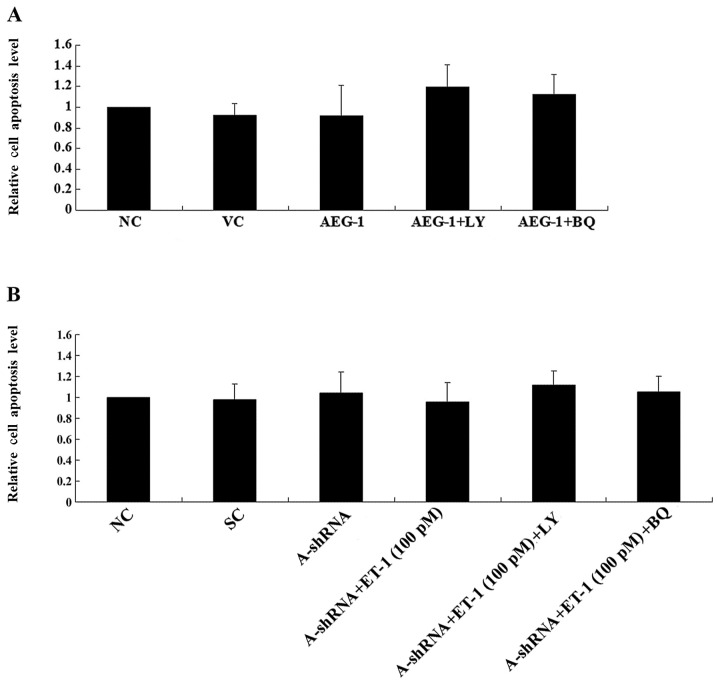
Cell apoptosis in Saos-2 and MG-63 cells under normal culture conditions. (A) Saos-2 and (B) MG-63 cells were under normal culture conditions for 8 h. The cell apoptosis rate was determined as the percentage of terminal deoxynucleotidyl transferase mediated nick-end labeling (TUNEL)-positive cells in total cells at 8 h. In Saos-2 cells, TUNEL assays were performed in normal control cells (NC), cells stably transfected with empty pcDNA3 vector (VC) and cells stably transfected with pcDNA3-AEG-1 expression vector (AEG-1) with or without LY294002 (50 *μ*M) or BQ123 (5 *μ*M). In MG-63 cells, TUNEL assays were performed in NC, cells stably transduced with scrambled control shRNA (SC), and cells stably transduced with AEG-1-shRNA (A-shRNA) with or without ET-1 (10 pM and 100 pM) treatment alone or ET-1 (100 pM) combined with LY294002 (50 *μ*M) or BQ123 (5 *μ*M). MG-63 cells treated with LY294002 (50 *μ*M) or BQ123 (5 *μ*M) alone were also analyzed. The cell apoptosis level is shown as the fold change relative to that of NC (designated as 1). ShRNA, short hairpin RNA.

**Figure 6.**
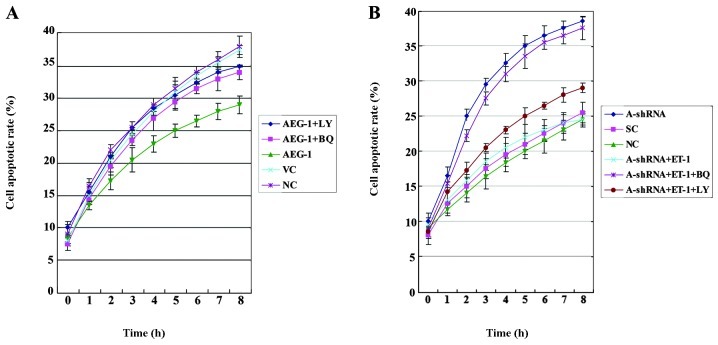
Cell apoptosis in Saos-2 and MG-63 cells treated with cisplatin. Saos-2 (A) and MG-63 (B) cells were treated with 10 nM of cisplatin for 8 h. In Saos-2 cells, terminal deoxynucleotidyl transferase mediated nick-end labeling (TUNEL) assays were performed in normal control cells (NC), cells stably transfected with empty pcDNA3 vector (VC), and cells stably transfected with pcDNA3-AEG-1 expression vector (AEG-1) with or without LY294002 (LY; 50 *μ*M) or BQ123 (BQ; 5 *μ*M). In MG-63 cells, TUNEL assays were performed in NC, cells stably transduced with scrambled control shRNA (SC), and cells stably transduced with AEG-1-shRNA (A-shRNA) with or without ET-1 (100 pM) treatment alone or ET-1 (100 pM) combined with LY294002 (50 *μ*M) (LY) or BQ123 (5 *μ*M) (BQ). MG-63 cells treated with LY (50 *μ*M) or BQ (5 *μ*M) alone were also analyzed. The cell apoptosis rate is shown as the percentage of TUNEL-positive cells in total cells. ShRNA, short hairpin RNA.

Both AEG-1 and ET-1/ETAR signaling have been demonstrated to promote tumor cell survival and chemoresistance (11,12). To investigate the effect of the interaction between AEG-1 and ET-1/ETAR signaling on OS cell survival, we examined the rate of cell apoptosis in the two cell lines that had been treated with 10 nM cisplatin, an apoptosis-inducing chemotherapeutic agent commonly used to treat OS. Overexpression or knockdown of AEG-1 in the presence or absence of ET-1 (100 pM) and/or LY294002 (50 *μ*M) or BQ123 (5 *μ*M) for ≤8 h did not significantly alter the rate of cell apoptosis in normal culture conditions (Fig. 5). In Saos-2 cells treated with cisplatin, over-expression of AEG-1 significantly decreased the rate of cell apoptosis compared with that of the controls, and was reversed by LY294002 or BQ123 (Fig. 6A). In MG-63 cells, knockdown of AEG-1 significantly increased cell apoptosis in the presence of cisplatin, and was reversed by treatment with exogenous ET-1. The rescue effect of ET-1 was completely blocked by BQ123 and partially blocked by LY294002 (Fig. 6B). Taken together, the results suggest that AEG-1 promotes OS cell survival against cisplatin partially, but significantly, through ET-1/ETAR, which functions downstream of PI3K.

## Discussion

As a multifunctional oncoprotein, AEG-1 has been demonstrated to enhance the aggressiveness of multiple types of human cancer, including OS (7,13,14). The ET-1/ETAR signaling pathway is a potential therapeutic target for the control of OS metastasis (9,10). In the present study, we explored the functional interaction between AEG-1 and ET-1/ETAR signaling in OS cells, and assessed its impact on OS cell invasion and survival.

We showed that Saos-2 cells exhibited a relatively low constitutive expression of AEG-1, while AEG-1 was amply expressed in MG-63 cells. Thus, overexpression and knockdown of AEG-1 were respectively performed in the two cell lines in order to utilise opposite approaches to the same study objective. Our results showed that AEG-1 regulated ET-1 expression at the transcriptional level in a PI3K-dependent manner in OS cells, which is concordant with the results of previous studies. A number of studies have demonstrated that AEG-1 triggers PI3K/Akt signaling in cancer cells (13,14). Additionally, Zhang *et al* found that AEG-1 regulated nuclear β-catenin accumulation in colorectal cell lines (15), while Sun *et al* demonstrated that nuclear β-catenin signaling regulated ET-1 transcription in a PI3K-dependent manner in prostate cancer cells (16). In the present study, we provide the first evidence that AEG-1 regulates ET-1 expression in a PI3K-dependent manner in OS cells. Further studies are required to address whether AEG-1 regulates ET-1 expression through nuclear β-catenin signaling.

Both AEG-1 and ET-1 have been demonstrated to promote OS cell invasion through MMP-2 (7,9). The present *in vitro* cell invasion assay results suggest that ET-1/ETAR signaling functions downstream of PI3K and primarily mediates the effect of AEG-1 on OS cell invasion. This is due to the fact that exogenous ET-1 was capable of restoring cell invasion and MMP-2 expression levels in MG-63 cells, in which AEG-1 had been knocked down, in the presence of a selective PI3K inhibitor (LY294002), but not in the presence of a selective ETAR inhibitor (BQ123). However, exogenous ET-1 only partially rescued cell survival against cisplatin-induced apoptosis in the presence of LY294002, in cells in which AEG-1 had been knocked down, suggesting that unlike that of cell invasion, alternative signaling pathways downstream of PI3K (other than ET-1/ETAR signaling) are involved in AEG-1-induced chemoresistance in OS cells.

Cisplatin elicits DNA repair mechanisms by crosslinking DNA, which in turn activates apoptosis when repair is not possible (17). It remains unclear whether the functional interaction between AEG-1 and ET-1/ETAR signaling is capable of impacting OS cell survival against other types of chemotherapy agents. Further studies with additional types of chemotherapy agents and OS cell lines are required.

In conclusion, we have demonstrated that AEG-1 regulates ET-1 expression at the transcription level in a PI3K-dependent manner in OS cells. Downstream of PI3K, ET-1/ETAR signaling primarily mediates the promoting effect of AEG-1 on OS cell invasion, likely through the upregulation of MMP-2 expression, while ET-1/ETAR signaling partially, but significantly, mediates the AEG-1-induced chemoresistance in OS cells. This study provides the first evidence of a functional link between AEG-1 and ET-1/ETAR signaling in OS cells, which adds novel insights into the molecular mechanism of OS metastasis and chemoresistance.

## References

[1] Ottaviani G, Jaffe N (2010). The epidemiology of osteosarcoma. Cancer Treat Res.

[2] Gorlick R, Anderson P, Andrulis I (2003). Biology of childhood osteogenic sarcoma and potential targets for therapeutic development: meeting summary. Clin Cancer Res.

[3] Wittig JC, Bickels J, Priebat D (2002). Osteosarcoma: a multi-disciplinary approach to diagnosis and treatment. Am Fam Physician.

[4] Su ZZ, Chen Y, Kang DC, Chao W, Simm M, Volsky DJ, Fisher PB (2003). Customized rapid subtraction hybridization (RaSH) gene microarrays identify overlapping expression changes in human fetal astrocytes resulting from human immunodeficiency virus-1 infection or tumor necrosis factor-alpha treatment. Gene.

[5] Liao WT, Guo L, Zhong Y, Wu YH, Li J, Song LB (2011). Astrocyte elevated gene-1 (AEG-1) is a marker for aggressive salivary gland carcinoma. J Transl Med.

[6] Lee SG, Su ZZ, Emdad L, Sarkar D, Fisher PB (2006). Astrocyte elevated gene-1 (AEG-1) is a target gene of oncogenic Ha-ras requiring phosphatidylinositol 3-kinase and c-Myc. Proc Natl Acad Sci U S A.

[7] Wang F, Ke ZF, Sun SJ (2011). Oncogenic roles of astrocyte elevated gene-1 (AEG-1) in osteosarcoma progression and prognosis. Cancer Biol Ther.

[8] Nelson J, Bagnato A, Battistini B, Nisen P (2003). The endothelin axis: emerging role in cancer. Nat Rev Cancer.

[9] Felx M, Guyot MC, Isler M (2006). Endothelin-1 (ET-1) promotes MMP-2 and MMP-9 induction involving the transcription factor NF-κB in human osteosarcoma. Clin Sci (Lond).

[10] Zhao Y, Liao Q, Zhu Y, Long H (2011). Endothelin-1 promotes osteosarcoma cell invasion and survival against cisplatin-induced apoptosis. Clin Orthop Relat Res.

[11] Yoo BK, Chen D, Su ZZ (2010). Molecular mechanism of chemoresistance by astrocyte elevated gene-1. Cancer Res.

[12] Rosanò L, Cianfrocca R, Spinella F (2011). Acquisition of chemoresistance and EMT phenotype is linked with activation of the endothelin A receptor pathway in ovarian carcinoma cells. Clin Cancer Res.

[13] Ying Z, Li J, Li M (2011). Astrocyte elevated gene 1: biological functions and molecular mechanism in cancer and beyond. Cell Biosci.

[14] Yoo BK, Emdad L, Lee SG (2011). Astrocyte elevated gene-1 (AEG-1): a multifunctional regulator of normal and abnormal physiology. Pharmacol Ther.

[15] Zhang F, Yang Q, Meng F, Shi H, Li H, Liang Y, Han A (2012). Astrocyte elevated gene-1 interacts with β-catenin and increases migration and invasion of colorectal carcinoma. Mol Carcinog.

[16] Sun P, Xiong H, Kim TH, Ren B, Zhang Z (2006). Positive inter-regulation between β-catenin/T cell factor-4 signaling and endothelin-1 signaling potentiates proliferation and survival of prostate cancer cells. Mol Pharmacol.

[17] Rosenberg B, VanCamp L, Trosko JE (1969). Platinum compounds: a new class of potent antitumour agents. Nature.

